# Enhanced aqueous phosphate removal using chitosan-modified zirconium-loaded cork biochar

**DOI:** 10.1038/s41598-025-14819-x

**Published:** 2025-08-10

**Authors:** Luiza Usevičiūtė, Artūras Kaklauskas, Vaidotas Danila, Tomas Januševičius

**Affiliations:** Civil Engineering Research Centre, Vilnius Gediminas Technical University, Saulėtekio Al. 11, 10223 Vilnius, Lithuania

**Keywords:** Phosphorus removal, Cross-linked chitosan-modified biochar, Zr(IV), Aqueous solutions, Isotherms, Kinetics, Cork biochar, Environmental sciences, Materials science

## Abstract

The adsorption of phosphate (PO_4_–P) is essential for controlling and reducing eutrophication. This study reports the synthesis of a new adsorbent material: Zr(IV)-loaded chitosan-modified used cork stopper biochar composite (CS–CBC–Zr) beads. The suitability of CS–CBC–Zr beads for PO_4_–P removal was assessed using the batch method. The effects of various parameters were investigated, including zirconium loading level, adsorbent dosage, pH, co-anions, contact time, and the initial concentration of PO_4_–P. The experimental data were thoroughly analyzed using adsorption kinetic and isotherm models. Virgin beads and P-adsorbed beads were characterized using FTIR, SEM, and XPS analyses. The results demonstrated that the combination of Zr coating and CS–CBC beads exhibited superior adsorption performance compared to individual CS–CBC beads. The CS–CBC–Zr beads exhibited a maximum adsorption capacity of 33.89 mg/g, as predicted using the Langmuir–Freundlich (Sips) model. The CS–CBC–Zr beads removed PO_4_–P with an efficiency of 95% at an initial pollutant concentration of 50 mg/L and reached adsorption equilibrium within 120 min of contact time, outperforming some comparable adsorbents. Moreover, the beads achieved excellent PO_4_–P removal performance over a wide pH range of 4–10, making them highly versatile. The experiment on the effect of coexisting anions demonstrated the excellent selectivity of CS–CBC–Zr for PO_4_–P. Phosphate adsorption on CS–CBC–Zr fitted well with the pseudo-second-order kinetic and Sips models. Kinetic data closely fitted the pseudo-second-order model, suggesting that adsorption was primarily governed by chemisorption.

## Introduction

The pollution of water bodies by nutrients, mainly due to an excess of phosphorus (P) and nitrogen (N), poses a significant risk to water quality in many countries. An excess of P and N in surface waters can lead to eutrophication, i.e., oxygen depletion caused by algal blooms, which affects both freshwater and marine ecosystems. As little as 0.02 mg/L of dissolved phosphate can trigger eutrophication by promoting algal blooms and the excessive consumption of dissolved oxygen, leading to severe environmental consequences ^1^. In Europe, 36% of rivers, 32% of lakes, and 81% of marine waters have been classified as eutrophic. Between 2016 and 2019, approximately 25% of Lithuanian rivers and a slightly lower percentage of lakes were also eutrophic ^2^. Phosphorus is the most common factor impairing water quality, and it is one of the primary causes of poor ecological status in water bodies. Currently, large amounts of P-containing products, such as detergents, washing powders, fertilizers, and pesticides, are widely used by humans, resulting in rising phosphorus levels in water bodies ^3^. On a global scale, the excess of P in the environment has already surpassed permissible limits, posing a significant threat to nature and the climate. This suggests that the world may soon reach peak P levels, where the demand for P exceeds the available global resources. Widespread eutrophication, combined with a potential P shortage, indicates that humanity may have already crossed critical global environmental boundaries.

Removing P during urban wastewater treatment is crucial to prevent its entry into water bodies. According to the European Union’s regulations, the maximum allowable total P concentration in treated wastewater is required to be 0.7 mg/L for wastewater treatment plants serving 10,000 to 150,000 population equivalents, and 0.5 mg/L is required for those serving more than 150,000 population equivalents ^4^. Currently, phosphates (PO_4_–P) from wastewater can be removed using various methods, including chemical precipitation ^5^, biological treatment, crystallization ^6^, reverse osmosis, and adsorption ^7^. However, the majority of these technologies present certain limitations. For instance, chemical precipitation is often constrained by the high cost of chemical additives, while biological removal relies on the elimination of phosphorus-rich sludge, a process associated with operational complexity, high sludge treatment costs, and the risk of secondary pollution. Among these approaches, adsorption stands out as one of the most effective and practical methods, even at low PO_4_–P concentrations ^8^. Its appeal lies in its operational simplicity, great efficiency and low cost ^9,10^. Moreover, many adsorbents are regenerable, making the process more sustainable.

A wide variety of materials have been explored for phosphate removal, including porous silica, zeolites, polymeric adsorbents, activated carbon, clay minerals, and biochar. Despite its popularity, activated carbon often exhibits poor phosphate adsorption due to its hydrophobicity and weak surface charge ^8^. Compared to activated carbon, biochar—produced from organic waste or agricultural biomass—offers a low-cost, resource-efficient alternative ^11^. Currently, various types of organic waste materials, such as agricultural residues (e.g., wheat straw, cow manure), sewage sludge, and cork powder, are used for biochar production, helping to reduce the global volume of waste. Agricultural waste and sewage sludge are widely available due to large-scale farming and continuous municipal wastewater treatment. In contrast, cork powder waste is more regionally concentrated, mainly in cork stopper-producing countries. Cork is a renewable and sustainable resource harvested from the outer bark of the cork oak tree. Its primary use is in the production of stoppers, which, after use, become waste products ^12^. Discarded cork stoppers can serve as a valuable feedstock for biochar production. Recent studies have increasingly focused on using cork powder, the primary byproduct of the cork industry, as a raw material for producing cork-based biochar ^13^. However, only a few studies have investigated the potential of using end-of-life cork stoppers for biochar production. Compared to the other types of organic waste, cork offers significant advantages as a biochar feedstock due to its unique cellular structure ^14^ and high surface area, which make it attractive for biochar production and the subsequent adsorption of water pollutants ^15^. Using biochar produced from used cork stoppers as a water remediation material reduces the cost of water and wastewater treatment while utilizing a recycled material. However, unmodified wood-based biochar exhibits poor phosphate adsorption capacity due to the abundance of electronegative carboxylic and phenolic groups, which create a negatively charged surface that repels phosphate anions. Therefore, modification is necessary to achieve effective phosphate removal ^16^.

Chitosan (CS) is the most important chemical derivative of chitin and the second most abundant natural biopolymer in the biosphere ^17^. CS is characterized by excellent adsorption properties, a high affinity for moisture, and an abundance of surface amino functional groups. Therefore, it is widely used for the removal of various contaminants, including heavy metal ions and PO_4_–P. Additionally, CS is an effective gel-forming agent ^18^ for the production of microspheres, making it easy to assemble and apply in large-scale wastewater treatment ^19^. When CS is combined with other materials as a surface modifier, the advantages of both materials can be enhanced ^20,21^. Zirconium-based composites have attracted particular attention as environmentally friendly functional adsorbents. These materials demonstrate high phosphate selectivity, low toxicity, and economic feasibility ^22^. As described in recent studies, Zr(IV) exhibits superior affinity toward phosphate compared to Al(III) and La(III) due to its higher positive charge and, therefore, stronger binding with phosphate anions, resulting in higher adsorption capacities ^23^. For example, Kumar and Viswanathan ^24^ investigated phosphate removal using clay and alginate composite beads. It was found that Zr(IV)-loaded alginate and clay composites had higher adsorption capacities than composites loaded with Mg(II), Ca(II), La(III), and Ce(III). Additionally, Zr compounds are known for their chemical stability, low toxicity, and minimal environmental risks, making them suitable for water treatment applications without harmful effects on aquatic life or public health ^25^. These advantages, combined with its compatibility with chitosan and cork biochar matrices, support the selection of Zr in this study.

Despite growing interest in Zr-based sorbents, a detailed understanding of their surface’s compositional change and structural transformation during the phosphate adsorption process remains limited, highlighting a key direction for future research. The novelty of this study lies in the use of low-cost, sustainable cork stopper biochar as a support matrix combined with zirconium and chitosan to enhance phosphate selectivity. Cork stopper biochar offers a cost-effective and renewable support material, while the incorporation of zirconium enhances phosphate selectivity, and chitosan serves as a natural polymer matrix that facilitates bead formation and provides functional groups for further interaction with phosphate ions. The performance of composite beads was evaluated across a wide pH range (4–10) and at varying initial PO_4_–P concentrations (5–500 mg/L). This study offers practical advantages, including the use of a waste-derived material and the investigation of adsorption performance of the composite under varying conditions. Nonetheless, additional synthesis steps—such as crosslinking and zirconium loading—may slightly increase preparation complexity, and further evaluation under real wastewater treatment conditions is needed to validate practical applicability of the material. The main objectives of this study are as follows: (1) to synthesize and characterize a CS–CBC–Zr composite derived from chitosan, cork stopper biochar, and Zr via scanning electron microscopy (SEM), Brunauer–Emmett–Teller (BET), X-ray photoelectron spectroscopy (XPS), and Fourier transform infrared spectroscopy (FTIR) analyses; (2) to assess the adsorption characteristics and potential mechanisms of PO_4_–P adsorption on CS–CBC–Zr from aqueous solutions.

## Materials and methods

### Materials

Chitosan (from shrimp shells, practical grade), glutaraldehyde solution (50 wt% in H_2_O), and zirconyl chloride octahydrate ([Zr_4_(OH)_8_(H_2_O)_16_]Cl_8_(H_2_O)_12_, 98%) were purchased from Sigma-Aldrich (Darmstadt, Germany). Phosphate stock solutions were prepared by dissolving potassium dihydrogen phosphate (KH_2_PO_4_) in distilled water. Champagne cork stoppers for biochar production were collected from local households. Sodium hydroxide (NaOH) and hydrochloric acid (HCl) were of analytical grade and purchased from POCH S.A and PPH Standard (Poland), respectively.

### Preparation of cork-based biochar and its composite with chitosan and zirconium(IV)

Cork biochar (CBC) was produced from spent wooden champagne cork stoppers through pyrolysis in a muffle furnace (E5CK-T) at 600 °C for a holding time of 2 h. To maintain anoxic conditions, the biomass was wrapped in aluminum foil. After pyrolysis, the biochar was cooled to room temperature, crushed, and sieved to a particle size of 100–200 µm for further use in the production of CS–CBC–Zr.

**Fig. 1:**
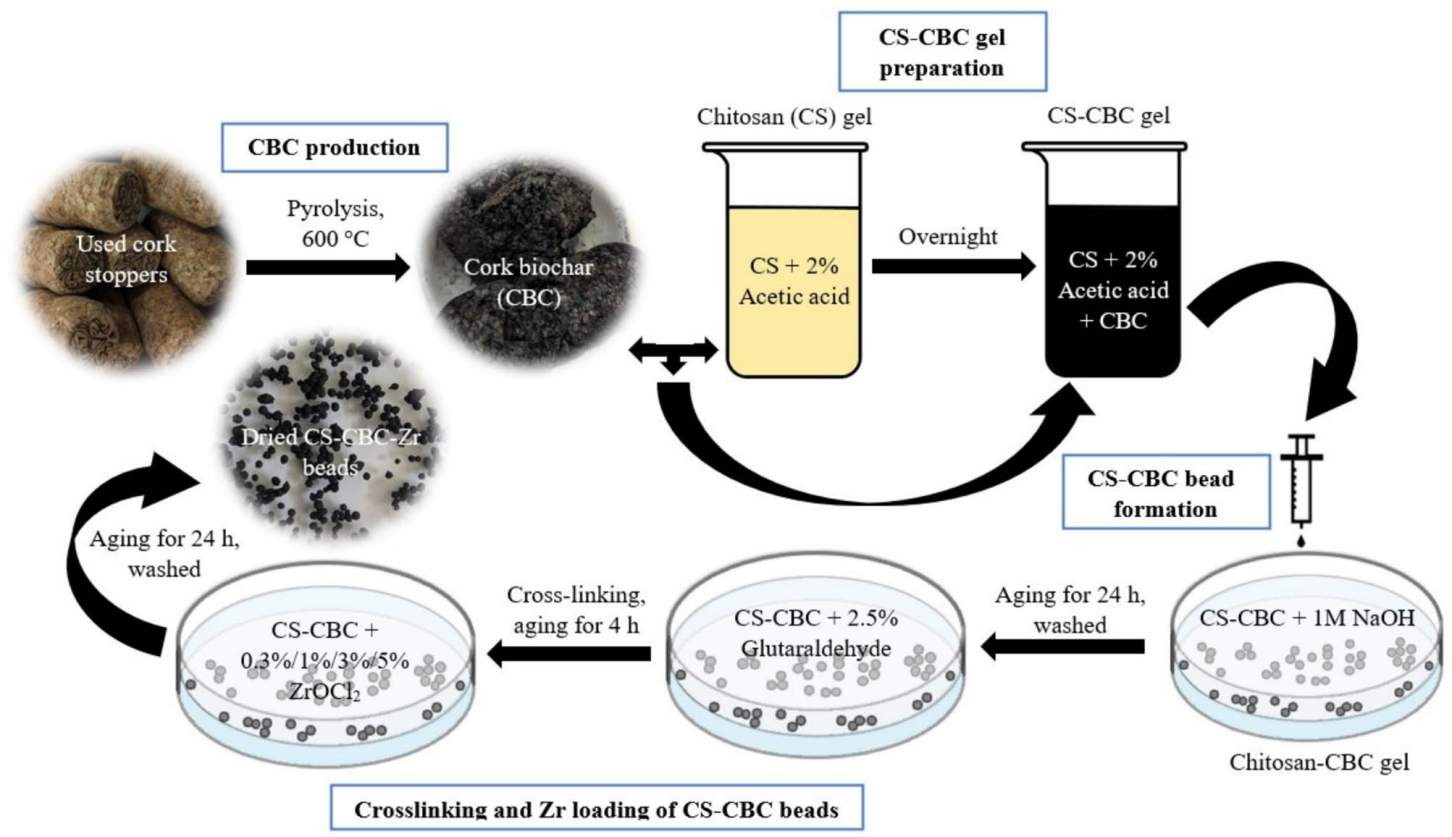
Schematic diagram of CS–CBC–Zr composite bead production.

CS–CBC–Zr was prepared following the method described by Banu et al. ^23^, with slight modifications. First, chitosan was used to modify CBC. A total of 10 g of chitosan was homogeneously dissolved in 500 mL of 2% acetic acid solution, and 5 g of biochar was mixed with the resulting solution. The mixture was stirred continuously for 5 h and left overnight to form a uniform dispersion of biochar and chitosan gel. Uniform CS–CBC beads were obtained by dropping the biochar–chitosan gel into a 1 M NaOH solution through a 2 mm diameter plastic hub needle (Zibo Eastmed Healthcare Products Co., Zhangdian district, China) and aging for 24 h. The beads were washed approximately 80 times with distilled water. Next, wet beads were soaked in a 2.5 wt% glutaraldehyde solution for 4 h ^26^. Glutaraldehyde was added to enhance the mechanical strength and chemical stability of chitosan, as it serves as a practical crosslinking agent ^11^. Chitosan undergoes a crosslinking reaction with glutaraldehyde, forming stable imine (–C=N–) bonds between polymer chains ^27^. Although the crosslinking process improves the structural stability of chitosan, it may also reduce its adsorption capacity for PO_4_–P due to the partial consumption of available adsorption sites. Therefore, further modifications of the CS–CBC composite are necessary to enhance its phosphate adsorption performance. Incorporating Zr(IV) ions into the CS–CBC composite can significantly improve its capacity to remove pollutants such as phosphate ^28^. Based on this approach, CS–CBC beads were soaked in ZrOCl_2_·8H_2_O solutions of varying concentrations (0.3%, 1%, 3%, and 5% w/v) for 24 h to immobilize Zr(IV) ions onto the CS–CBC beads. The CS–CBC–Zr beads were then washed twice with distilled water to remove excess ions. Finally, the resulting adsorbent was dried at room temperature (≈ 25 $^\circ$C) to obtain CS–CBC–Zr composite beads. The preparation process of CS–CBC–Zr beads is shown in Fig. 1.

### Characterization of CS–CBC–Zr beads

The physicochemical properties of the prepared CS–CBC–Zr composite were analyzed using SEM, BET, FTIR and XPS techniques. The surface morphology of the composite before and after PO_4_–P adsorption was evaluated using a scanning electron microscope (SEM, TM4000, Hitachi, Japan). The average diameter of the CS–CBC–Zr beads was measured and calculated using the ImageJ program (National Institute of Health, USA). The specific surface area, pore volume, and pore size of CBC and CS–CBC–Zr beads were determined using the BET method with N_2_ adsorption–desorption at 77 K on a NOVA touch 2LX device (Quantachrome, Boynton Beach, FL, USA). An FTIR spectrometer (Bruker, Invenio) was used to identify the functional groups of CS, CBC, zirconium oxychloride, and the composite before and after adsorption at wavelengths ranging from 500 to 4000 cm^−1^ via the KBr pellet method. XPS analysis of the composite beads was performed using a Kratos Axis Supra spectrometer with a monochromatic Al Kα source. The acquired data were analyzed using CasaXPS software (version 2.3.23rev1.1R).

### Batch adsorption studies

A stock solution containing 500 mg/L of P was prepared by dissolving KH_2_PO_4_ in distilled water, and the desired concentrations were obtained by diluting the stock solution. Batch adsorption experiments were conducted using a rotary shaker (Rotoshake RS12, Gerhardt GmbH, Germany). Phosphate concentrations were determined via molybdenum blue method at a wavelength of 880 nm using a UV spectrophotometer (UV-1800, Shimadzu, Japan). The intensity of the blue color is directly proportional to the PO_4_–P concentration. For each test, samples were filtered through 0.45 µm membrane filters. All experiments were performed under the following conditions unless stated otherwise: adsorption time: 30 min; initial solution pH: natural (except for experiment investigating effect of initial pH); rotation speed: 12 rpm; initial PO_4_–P concentration: 50 mg/L (except for the isotherm study); PO_4_–P solution volume: 50 mL; adsorbent dose: 0.45 g. After adsorption, the PO_4_–P removal efficiency (R, %) was calculated using Eq. (1):

1 $$R=\left(\frac{{C}_{0}-{C}_{e}}{{C}_{0}}\right)\times 100,$$

where *R* is the PO_4_–P removal efficiency (%), and *C*_*0*_ and *C*_*e*_ are the initial and equilibrium PO_4_–P concentrations (mg/L), respectively.

The equilibrium adsorption capacity (*q*_*e*_, mg/g) was calculated using Eq. (2):

2 $${q}_{e}=\left(\frac{{C}_{0}-{C}_{e}}{m}\right)\times V,$$

where *q*_*e*_ represents the PO_4_–P adsorption capacity at equilibrium time (mg/g); *V* is the volume of the solution (L); and *m* is the mass of the adsorbent (g).

Adsorption capacities at different time intervals *t* (*q*_*t*_, mg/g) were calculated using Eq. (3):

3 $${q}_{t}=\frac{\left({C}_{0}-{C}_{t}\right)}{m}\times V,$$

where *C*_*0*_ and *C*_*t*_ denote the initial PO_4_–P concentration and the PO_4_–P concentration at time *t* (mg/L), respectively; *V* represents the volume of the solution (L); and *m* is the mass of the adsorbent (g).

#### Effect of ZrOCl_2_·8H_2_O concentration

Zirconium content is an important parameter that influences the adsorption performance of various adsorbents ^16^. Therefore, the effect of ZrOCl_2_·8H_2_O concentration on PO_4_–P adsorption was studied using previously prepared CS–CBC–Zr beads immersed in different ZrOCl_2_·8H_2_O solution concentrations (0.3, 1, 3, and 5%). In total, 0.1 g of CS–CBC–Zr beads was added to 100 mL bottles containing 50 mL of a 50 mg/L PO_4_–P solution at natural pH (≈ 5–6) and shaken in a rotary shaker for 30 min. For comparison, two other adsorbent types without Zr were studied: CS–CBC (without a crosslinker) and CS–CBC (with a crosslinker). The CS–CBC–Zr beads that demonstrated most efficient PO_4_–P removal were selected for further experiments.

#### Effect of adsorbent dosage

To evaluate the effect of adsorbent dosage on the adsorption, different dosages of CS–CBC–Zr ranging from 0.025 to 0.550 g (0.025, 0.050, 0.075, 0.100, 0.125, 0.150 g, 0.250 g, 0.350 g, 0.450 g, and 0.550 g) were added to a 50 mg/L PO_4_–P solution at natural pH levels. The bottles containing the solution were placed in a rotary shaker at 23 ± 2 $^\circ$C for 30 min.

#### Effect of initial aqueous solution pH on PO_4_–P adsorption and point of zero charge

To study the effect of solution pH on PO_4_–P adsorption, 0.45 g of CS–CBC–Zr was added to 50 mL of PO_4_–P solution with an initial concentration of 50 mg/L over a pH range of 2–12 (2, 4, 6, 8, 10, and 12). The pH values of the solutions were adjusted using 0.1 M HCl and 0.1 M NaOH. Experiments were conducted for 30 min. The point of zero charge (pH_PZC_) of the CS–CBC–Zr composite beads was determined using the method described by Manyatshe et al. ^29^. We transferred 0.1 M NaCl solutions (50 mL) into a series of 100 mL glass bottles. The initial pH (pH_i_) of the solutions was then adjusted to 2–12 using 0.1 M HCl or 0.1 M NaOH. Next, 0.45 g of CS–CBC–Zr beads was added to the solutions, which were shaken for 24 h, after which the final pH (pH_f_) was recorded. The difference between the initial and final pH values (∆pH = pH_i_–pH_f_) was plotted against the pH_i_ to determine pH_PZC_, where ∆pH = 0, indicating the point of zero charge of the sample ^30^.

#### Effect of coexisting ions

Phosphate-polluted wastewater usually contains several other anions, such as nitrate (NO_3_^–^), carbonate (CO_3_^2–^), chloride (Cl^–^), and sulphate (SO_4_^2–^), which can compete with PO_4_–P ions for adsorption sites on CS–CBC–Zr. To determine the influence of coexisting anions, 0.45 g of CS–CBC–Zr was added to 50 mL of the solution containing 50 mg/L PO_4_–P, along with typical anions such as NO_3_^–^, CO_3_^2–^, Cl^-^, and SO_4_^2–^ and a mixture of all anions ^31^. The concentrations of these co-anions in the solutions varied (0, 50, 100, and 200 mg/L) and were prepared using NaNO_3_, Na_2_CO_3_, NaCl, and Na_2_SO_4_ salts ^32^. Different concentrations of competing ions were selected to reflect the natural variability of ion concentrations in water sources ^33^. The solution’s pH remained natural.

#### Phosphate adsorption kinetics

Phosphate adsorption kinetics on CS–CBC–Zr were assessed by adding 0.45 g of CS–CBC–Zr to 50 mL of PO_4_–P solution at a concentration of 50 mg/L. The supernatant was collected and analyzed at different time intervals (5–240 min) (5, 10, 15, 20, 25, 30, 40, 50, 60, 120, and 240 min). The adsorption capacity at time *t* was calculated using Eq. (3). Adsorption kinetic experiments were conducted at natural pH levels.

#### Phosphate adsorption isotherms

The adsorption isotherms of CS–CBC–Zr were studied by adding 0.45 g of CS–CBC–Zr to 50 mL of PO_4_–P solutions with initial concentrations ranging from 5 to 500 mg/L (5, 10, 25, 50, 100, 150, 200, 300, and 500 mg/L) at a natural pH level. The bottles containing the solution were placed in a rotary shaker and rotated at 23 ± 2 $^\circ$C for 30 min. The solutions were then filtered, and the PO_4_–P concentration was determined using a UV spectrophotometer. The adsorption capacity at equilibrium was calculated using Eq. (2).

#### Kinetic models and isotherm models

The experimental results were fitted using non-linear kinetic models (pseudo-first-order, pseudo-second-order, and intraparticle diffusion) and non-linear isotherm models [Langmuir, Freundlich, Langmuir–Freundlich (Sips), and Dubinin–Radushkevich (D–R)]. Non-linear regression analysis is often the preferred method for determining isotherm and kinetic parameters ^34^. The Langmuir model describes a homogeneous adsorption process involving monolayer adsorption, whereas the Freundlich model represents a heterogeneous surface or multilayer adsorption ^35^. The Sips model, also known as the Langmuir–Freundlich model, is a hybrid of the Langmuir and Freundlich models. It is a semi-theoretical approach that integrates the advantages of both models, reducing to the Freundlich model at low concentrations and approaching the Langmuir model at higher concentrations ^36,37^. Additionally, the dimensionless separation factor (*R*_*L*_) was calculated to determine the key parameter of the Langmuir isotherm. Based on this value, adsorption can be classified as favorable (0 < *R*_*L*_ < 1), linear (*R*_*L*_ = 1), irreversible (*R*_*L*_ = 0), or unfavorable (*R*_*L*_ > 1) ^38^. Unlike the Langmuir isotherm, the D–R model is more versatile, as it does not rely on the assumption of a homogeneous surface or uniform sorption energy. It was applied to estimate the mean free energy *E* (kJ/mol) of adsorption, which helps determine the underlying sorption mechanism—whether it involves physical adsorption (*E* < 8 kJ/mol), ion exchange (8 kJ/mol < *E* < 16 kJ/mol), or chemical adsorption (*E* > 16 kJ/mol) ^39^. All kinetic and isotherm models used in this study, along with their mathematical expressions, are listed in Table 1.

**Table 1:** Expressions for the kinetic and isotherm models used in this study, along with their experimentally determined parameter values for PO_4_-P adsorption on CS-CBC-Zr beads.

Models	Expressions	Parameters	References
Adsorption kinetics			
Pseudo-first-order	${q}_{t}={q}_{e}(1-{e}^{{-k}_{1}t})$, where *q*_*e*_ (mg/g) represents the adsorbed amount of P at adsorption equilibrium; *q*_*t*_ (mg/g) represents the adsorbed amount of P at adsorption time, *t*; *k*_*1*_ (min^−1^) represents the reaction rate constant	*q*_*e*_ = 5.14815 (mg/g) *k*_*1*_ = 0.07239	^17,32,40^
Pseudo-second-order	${q}_{t}=\frac{{q}_{e}^{2}{k}_{2}t}{1+{q}_{e}{k}_{2}t}$, where *k*_*2*_ (g × mg × min^−1^) represents the reaction rate constant	*q*_*e*_ = 5.72519 (mg/g) *k*_*2*_ = 0.01845	^17,32^
Intraparticle diffusion	${q}_{t}={K}_{idiff}{t}^\frac{1}{2}+C$, where *K*_*idiff*_ (g × mg × min^−1^) represents the reaction initial rate constant for the intraparticle diffusion model; *C* is a constant related to boundary layer thickness ${K}_{idiff}=\frac{6\times {q}_{e}}{r\times {\left(\frac{D}{\pi }\right)}^\frac{1}{2}}{K}_{idiff}{t}^\frac{1}{2}$, where *r* (cm) is a radius of adsorbent particle; *D* (cm^2^/min) is an intraparticle diffusion coefficient	*K*_*idiff*_ = 0.30872 *C* = 1.9901	^17,32^
Adsorption isotherms			
Langmuir	${q}_{e}=\frac{{q}_{max}{K}_{L}{C}_{e}}{1+{K}_{L}{C}_{e}}$, where *q*_*max*_ (mg/g) is the maximum amount of P taken up by the adsorbent; *K*_*L*_ (l/mg) is the energy factor ${R}_{L}=\frac{1}{1+{K}_{L}{C}_{0}}$, where *R*_*L*_ is a separation factor (equilibrium parameter); *C*_*0*_ is the initial concentration of adsorbate in the solution, mg/L	*q*_*max*_ = 35.94 *K*_*L*_ = 0.045 *R*_*L*_ = 0.816 (for 5 mg/L) *R*_*L*_ = 0.308 (for 50 mg/L) *R*_*L*_ = 0.043 (for 500 mg/L)	^17,36,41,42^
Freundlich	${q}_{e}={K}_{F}{C}_{e}^\frac{1}{n}$, where *K*_*F*_ ((mg/g)(l/mg)^1/*n*^) and *n* are constants related to the sorption capacity and intensity	*K*_*F*_ = 3.874 1/*n* = 0.415	^17,36,41,42^
Sips	${q}_{e}=\frac{k{q}_{max}{C}_{e}^\frac{1}{n}}{1+k{C}_{e}^\frac{1}{n}}$, where *k* (l/mg) is the constant; 1/*n* is the heterogeneity factor	*k* = 0.038 1/*n* = 0.889 *n* = 1.124 *q*_*max*_ = 33.89	^36,42,43^
Dubinin–Radushkevich	${q}_{e}={q}_{max}\text{exp}\left(-\beta {\upvarepsilon }^{2}\right)$, where *q*_*max*_ is the theoretical maximum adsorption capacity (mg/g), *β* is coefficient related to adsorption energy (mol^2^/kJ^2^), and *ε* is the Polanyi potential (kJ/mol) $\varepsilon =RTln(\frac{{C}_{s}}{{C}_{e}})$, where *R* is universal gas constant (0.008314 kJ/mol/K), *T* is absolute temperature (K), *C*_*s*_ is saturation solubility (mg/l), *C*_*e*_ is equilibrium concentration of adsorbate (mg/l) $E=\frac{1}{\sqrt{2\beta }}$, where *E* is the mean free energy of adsorption (kJ/mol)	*E* = 11.24 *q*_*max*_ = 112.6 *β* = 0.004	^44–46^

### Desorption and regeneration studies

The reusability of CS–CBC–Zr were determined through adsorption–desorption cycle experiments. The CS–CBC–Zr beads were regenerated using 2 M NaOH as a desorbent solution. Before regeneration, the most suitable desorption solution (NaOH or NaCl) and its optimal concentration (0.1 M, 0.5 M, 1 M, or 2 M) were identified based on the highest obtained desorption efficiency. The desorption efficiency (%) was calculated using Eq. (4) ^35^. In total, 0.45 g of the PO_4_^3−^-loaded samples from the previous adsorption isotherm study (initial PO_4_–P concentration: 50 mg/L) was immersed in the desorbent solution and shaken for 1 h at room temperature to achieve desorption ^16,47^. After desorption, the concentration of desorbed PO_4_–P ions in the solution was analyzed using a spectrophotometer, as previously described. The adsorbent was then rinsed with deionized water to remove the residual NaOH solution, achieving a neutral pH, and dried at room temperature; the regenerated CS–CBC–Zr beads were then reused for adsorption. The regeneration efficiency (%) of the composite beads was calculated using Eq. (5)^48^:

4 $$Desorption efficiency (\%)=\frac{{m}_{d}}{{m}_{0}}\times 100,$$

5 $$Regeneration efficiency (\%)=\frac{{q}_{ar}}{{q}_{0}}\times 100,$$

where *m*_*d*_ denotes the amount of PO_4_–P desorbed from the adsorbent (mg/g), *m*_*0*_ denotes the amount of PO_4_–P remaining on the adsorbent after adsorption (mg/g), *q*_*ar*_ denotes the adsorption capacity after regeneration (mg/g), and *q*_*0*_ denotes the initial adsorption capacity (mg/g).

### Statistical analysis

All experiments were conducted in duplicate, and the results were expressed as mean ± standard deviation (SD) to ensure data reliability and accuracy. The parameters of the kinetic and isotherm models were calculated using Origin 2019b 64-bit Academic software (OriginLab Corporation, USA). Model fitting was performed using the Levenberg–Marquardt iteration algorithm ^49^. The coefficient of determination (R^2^) and various error functions were used as criteria for selecting the best kinetic and isotherm models. The optimal kinetic and isotherm models were identified through statistical analysis, considering several key metrics, including R^2^, the root mean square error (RMSE), the hybrid fractional error function (HYBRID), Marquardt’s percent standard deviation (MPSD), and the chi-square test (x^2^). A model was considered a better fit when the R^2^ value was higher, and the error function values were lower. All error calculations were performed using Microsoft Excel. The types of error functions, along with the coefficient of determination and their mathematical expressions, are provided in Table 2.

**Table 2:** Types of error functions and their mathematical expressions.

Type of error	Abbreviation	Expression	References
Sum of square errors	SSE	$SSE=\sum_{i=1}^{n}{({q}_{e,cal}-{q}_{e,exp})}_{i}^{2}$	^50^
Hybrid fractional error	HYBRID	$HYBRID=\frac{100}{n-p}\sum_{i=1}^{n}{\left[\frac{{\left({q}_{e,cal}-{q}_{e,exp}\right)}_{i}^{2}}{{q}_{e,exp}}\right]}_{i}$	
Marquardt’s percent standard deviation	MPSD	$MPSD=100\sqrt{{\frac{1}{n-p}\sum_{i=1}^{n}\left[\frac{{\left({q}_{e,cal}-{q}_{e,exp}\right)}_{i}^{2}}{{q}_{e,exp}}\right]}_{i}}$	
Root mean square error	RMSE	$RMSE=\sqrt{\frac{1}{n}\sum_{i=1}^{n}{\left({q}_{e,cal}-{q}_{e,exp}\right)}_{i}^{2}}$	
Chi-square test	${\text{x}}^{2}$	${x}^{2}=\sum_{i=1}^{n}{\left[\frac{{\left({q}_{e,cal}-{q}_{e,exp}\right)}_{i}^{2}}{{q}_{e,exp}}\right]}_{i}$	
Coefficient of determination	R^2^	${R}^{2}=1-\frac{\sum {\left({q}_{e,exp}-{q}_{e,cal}\right)}^{2}}{\sum {\left({q}_{e,exp}-{q}_{e,mean}\right)}^{2}}$	^51^

## Results and discussion

### Characterization of adsorbent

#### Surface morphology

**Fig. 2:**
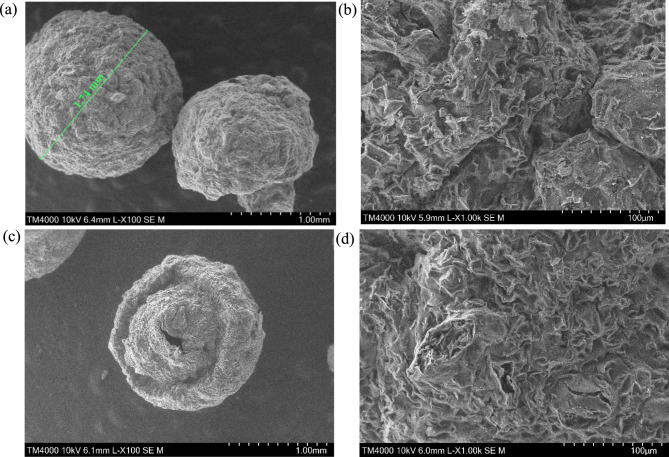
SEM images of CS–CBC–Zr composite beads before (**a**,**b**) and after (**c**,**d**) PO_4_–P adsorption.

The SEM images of CS–CBC–Zr beads before and after PO_4_–P adsorption are shown in Fig. 2. A 100 × magnification with a 1 mm scale bar was used to investigate bead morphology, while a 1000 × magnification with a 100 µm scale bar was used to examine the surface structure, as shown in Fig. 2a–d. As observed in Fig. 2a, the composite beads were successfully fabricated with an approximate grain size of 1.74 mm. Moreover, the SEM images of the composite beads revealed a spherical shape, although the material was heterogeneous with many irregularities and a rough surface. A rougher surface increases the possibility of contaminants adhering to the beads ^52^. Additionally, the surface of the beads resembled a honeycomb pattern, which is attributed to a network of layered wavy wrinkles with numerous pores visible as voids within the structure (Fig. 2b). These characteristics are consistent with CBC, and they were highlighted in previous studies ^15,33,53^. The visible voids in the SEM images align with the porous nature of biochar derived from cork materials. The observed rough surface morphology likely resulted from the integration of chitosan and biochar. Morphological analyses suggested that the composite material possessed surface characteristics suitable for efficient pollutant adsorption. Figures 2c,d show that after PO_4_–P adsorption, the beads maintained a similar morphology to fresh beads. However, in some areas, beads with adsorbed phosphorus exhibited small cracks. After PO_4_–P adsorption, some pores collapsed or were filled with adsorbed PO_4_–P.

#### FTIR analysis of adsorbents before and after adsorption

**Fig. 3:**
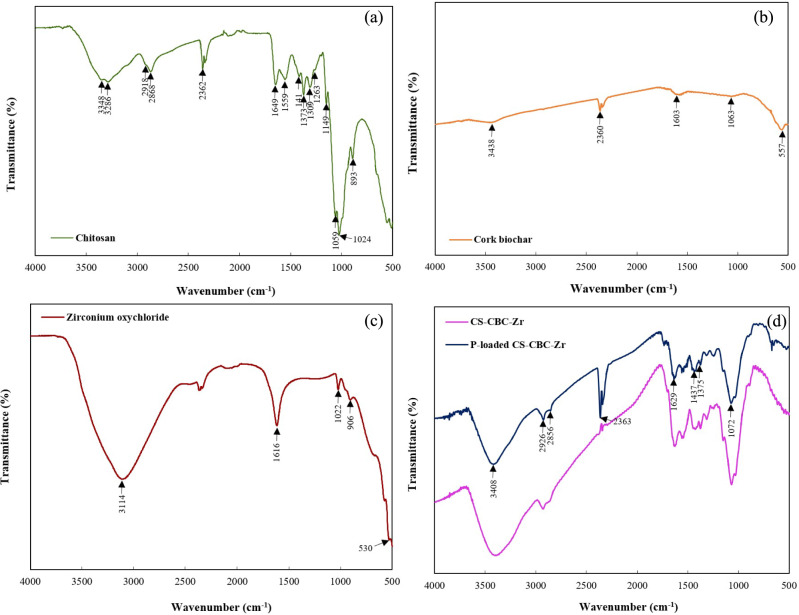
FTIR spectra of (**a**) chitosan, (**b**) CBC, (**c**) zirconium oxychloride, and (**d**) CS–CBC–Zr beads before and after PO_4_–P adsorption.

The FTIR spectra of CS are shown in Fig. 3a. The broad band at 3348 cm⁻^1^ corresponds to the stretching vibrations of O–H and N–H groups, indicating the presence of amino and hydroxyl groups on the CS surface ^54^. The vibrational peaks at 2918 cm⁻^1^ and 2868 cm⁻^1^ were attributed to the stretching vibrations of C–H and C–H_2_^23,47^. A peak at 1649 cm⁻^1^ corresponds to the C=O stretching vibration of the amide (–NH_2_) groups ^55^. Bands at 1417 cm⁻^1^ and 1373 cm⁻^1^ were attributed to the C–CH_3_ group ^56^. The 1263 cm⁻^1^ band corresponds to the amide III group. Absorption bands at 1149 cm⁻^1^, 1059 cm⁻^1^, and 1024 cm^−1^ are characteristic of the saccharide structure of chitosan ^57^. The 1149 cm⁻^1^ band is attributed to the asymmetric stretching of the C–O–C bridge, while the bands near 1059 cm⁻^1^ and 1024 cm⁻^1^ correspond to the C–O stretching vibrations.

The FTIR spectra of CBC can be seen in Fig. 3b. In the spectrum of CBC, weak bands were attributed to the primary components of cork cell walls, mainly residual lignin remaining after thermal degradation ^58^. A broad and weak band at 3438 cm⁻^1^ indicated the presence of O–H groups. The distinctive peak of lignin was observed at 1603 cm⁻^1^, corresponding to the vibration of C=C bonds within aromatic rings ^59^.

The FTIR spectra of zirconium oxychloride can be seen in Fig. 3c. Five peaks were detected for zirconium at 3114 cm⁻^1^, 1616 cm⁻^1^, 1022 cm⁻^1^, 906 cm⁻^1^, and 530 cm⁻^1^. The prominent band at 3114 cm⁻^1^ was attributed to the stretching vibrations of O–H bonds, originating from water molecules and hydroxyl groups ^30^. Additionally, a peak at 1616 cm⁻^1^ was attributed to the H–O–H bending vibration. A vibration at 530 cm⁻^1^ aligns with the Zr–O–Zr stretching mode, while vibrations at 906 cm⁻^1^ and 1022 cm⁻^1^ correspond to Zr=O stretching ^60,61^.

The FTIR spectra of the original CS–CBC–Zr composite beads and those after PO_4_–P adsorption are shown in Fig. 3d. Before PO_4_–P adsorption, CS–CBC–Zr exhibited strong absorption peaks at 3408 cm⁻^1^, 2926 cm⁻^1^, 2856 cm⁻^1^, 1629 cm⁻^1^, and 1437 cm⁻^1^, corresponding to O–H, C–H, N–H, and Zr–OH functional groups (Fig. 3d). A strong and broad band in the 3600–3100 cm^−1^ region indicates the stretching vibration of the O–H bond ^30,62^. The bands at 2926 cm⁻^1^ and 2856 cm⁻^1^ were attributed to the C–H stretching vibration of methylene and methyl groups ^63^. Compared to pristine CBC, the presence of a 1437 cm⁻^1^ band in the CS–CBC–Zr spectrum indicates the presence of surface hydroxyl (Zr–OH) groups ^64^. The O–H band at 1437 cm⁻^1^ and the C–H band at 1375 cm⁻^1^ weakened after PO_4_–P adsorption on CS–CBC–Zr beads. Additionally, a characteristic peak at 1072 cm⁻^1^ decreased in intensity after adsorption, suggesting that surface hydroxyl groups were replaced by adsorbed PO_4_–P. A slight broadening of the 1072 cm⁻^1^ band was observed in phosphate-adsorbed CS–CBC–Zr, indicating the presence of PO_4_–P ^62^. FTIR analysis clearly showed that CS–CBC–Zr beads contained a greater number of reactive oxidative functional groups compared to CBC, making them more likely to interact with PO_4_–P ions.

#### XPS analysis of adsorbents before and after adsorption

**Fig. 4:**
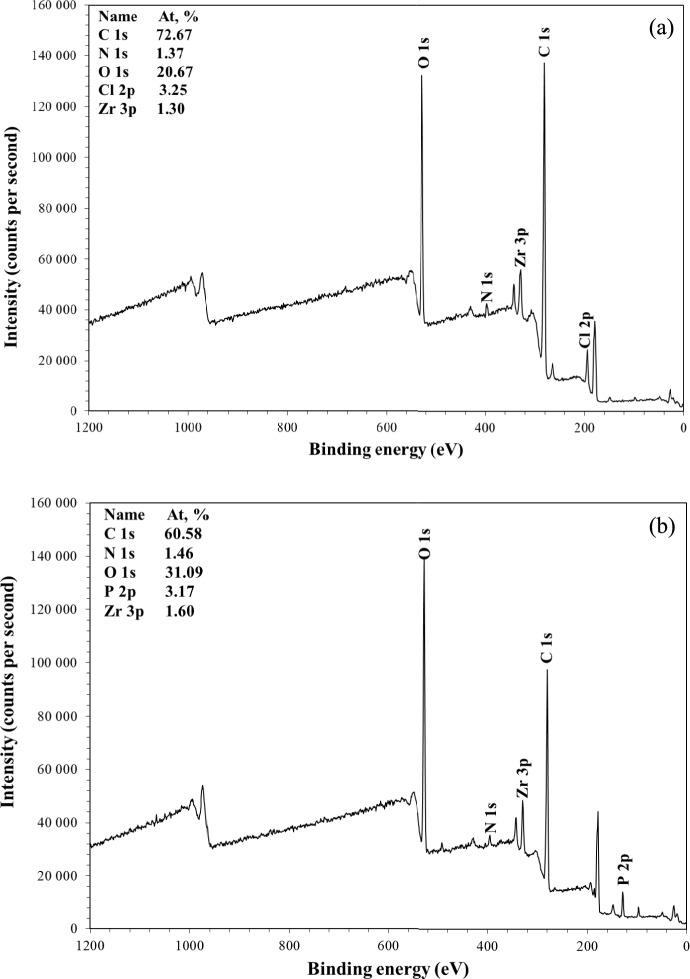
XPS spectra of CS–CBC–Zr composite beads before (**a**) and after (**b**) PO_4_–P adsorption.

The XPS spectra of CS–CBC–Zr composite beads before and after phosphorus adsorption are shown in Fig. 4. From the results, it can be confirmed that Zr(IV) was efficiently loaded on the surface of CS–CBC–Zr beads. Other elements that could be identified from the XPS spectroscopy of CS–CBC–Zr composite beads are C, O, N, and Cl. The XPS spectra of composite beads obtained after PO_4_–P adsorption show a new peak at 133.78 eV, corresponding to the P 2p spectra. This confirms that phosphate was successfully adsorbed on the surface of CS–CBC–Zr. The XPS results also suggest that phosphate adsorption occurred through an ion exchange, in which chloride ions were displaced. For CS–CBC–Zr beads obtained after PO_4_–P adsorption, it was clear that the intensity of the Cl 2p peak decreased in the XPS spectrum.

#### BET analysis

The textural properties of CBC and CS–CBC–Zr composite beads are presented in Table 3. The BET surface area, total pore volume, and average pore diameter of CBC were found to be 358 m^2^/g, 0.177 cm^3^/g, and 0.992 nm, respectively, while those of CS–CBC–Zr beads were 11.8 m^2^/g, 0.0199 cm^3^/g, and 2 nm, respectively. The surface area of the composite beads was approximately 30 times lower than that of the raw biochar. This reduction could be attributed to two main factors. Firstly, the crosslinking between CBC and chitosan likely blocked some of the pores within the biochar structure, resulting in a denser and more compact material ^65^. Secondly, the formation of composite beads led to an increase in overall particle size, which resulted in a reduction in the specific surface area.

**Table 3:** Specific surface area and pore properties of cork biochar (CBC) and chitosan-modified zirconium-loaded CBC composite beads (CS–CBC–Zr).

Material	Specific surface area (m^2^/g)	Total pore volume (cm^3^/g)	Average pore diameter (nm)
CBC	358	0.177	0.992
CS–CBC–Zr	11.8	0.0199	2

Table 4 presents a comparison of the BET surface area of CS–CBC–Zr beads with those of other adsorbents reported in previous studies. As shown in this table, the specific surface area of CS–CBC–Zr beads was higher compared to other Zr-loaded chitosan beads. The relatively larger surface area of ​​these beads confirms their potential for PO_4_–P removal.

**Table 4:** Comparison of the surface area of different chitosan beads.

Chitosan composite beads	Specific surface area (m^2^/g)	References
Zr encapsulated chitosan quaternized beads	0.35	^47^
Zr-loaded cross-linked chitosan beads	2.02	^66^
Zr-loaded cross-linked chitosan beads prepared by silica dissolution method	9.04	^66^
Chitosan modified Zr-loaded CBC beads	11.8	This work

It can be seen that the surface area of used cork stopper biochar, prepared at 600 °C, was 358 m^2^/g in this study. Comparing the specific surface area of CBC produced in this study with values reported in previous studies, this value falls within the range of 321.6–447.86 m^2^/g ^15,67^. These results indicate that CBC, with its large surface area, can be used in wastewater treatment. The well-developed pore structure and high surface area significantly enhance the adsorptive properties of biochar by offering available space for contaminant adsorption ^68^.

### Effect of influencing factors on phosphate adsorption

#### Effect of ZrOCl_2_·8H_2_O concentration

**Fig. 5:**
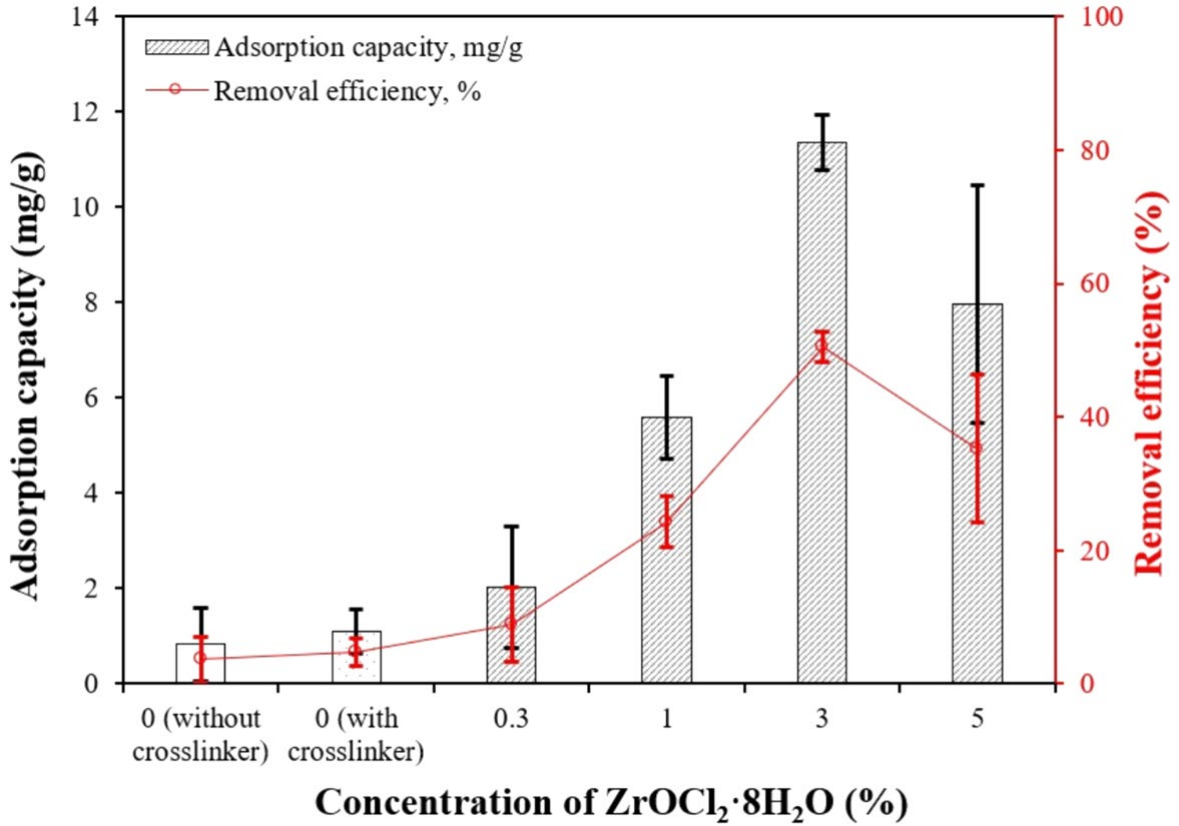
Effect of ZrOCl_2_·8H_2_O concentration on PO_4_–P adsorption (initial PO_4_–P concentration = 50 mg/L; adsorbent dosage = 0.1 g; solution volume = 50 mL; initial pH = 5.151; contact time = 30 min; solution temperature = 24.2 °C), mean ± SD.

The effect of ZrOCl_2_·8H_2_O concentration on adsorption was investigated using a 50 mg/L PO_4_–P solution without pH adjustments, and the results are shown in Fig. 5. The CS–CBC beads (without Zr) exhibited much lower PO_4_–P removal efficiency compared to those loaded with Zr. When comparing the effect of the crosslinking agent (glutaraldehyde) on PO_4_–P removal, it was observed that its influence was not significant, as both adsorbents (with and without crosslinker) had similar adsorption capacities (around 1 mg/g). It is clear that loading CS–CBC beads with Zr resulted in more effective phosphorus removal than composite beads without Zr. Both adsorption capacity and PO_4_–P removal efficiency increased significantly as the ZrOCl_2_·8H_2_O concentration increased from 0 to 3%. The highest adsorption capacity (11.4 mg/g) and removal efficiency (50.6%) of CS–CBC–Zr were observed at a ZrOCl_2_·8H_2_O concentration of 3%. At ZrOCl_2_·8H_2_O concentrations above 3%, it is possible that a smaller amount of Zr was complexed with the functional groups on the CS–CBC surface, leading to a lower adsorption capacity. Additionally, composites with lower Zr content may result in higher Zr utilization efficiency and greater accessibility to phosphorus. Considering both removal efficiency and the cost of ZrOCl_2_·8H_2_O, for subsequent adsorption experiments, CS–CBC–Zr beads were prepared using a 3% ZrOCl_2_·8H_2_O solution (equivalent to 0.093 mol/L).

#### Effect of CS–CBC–Zr dosage

**Fig. 6:**
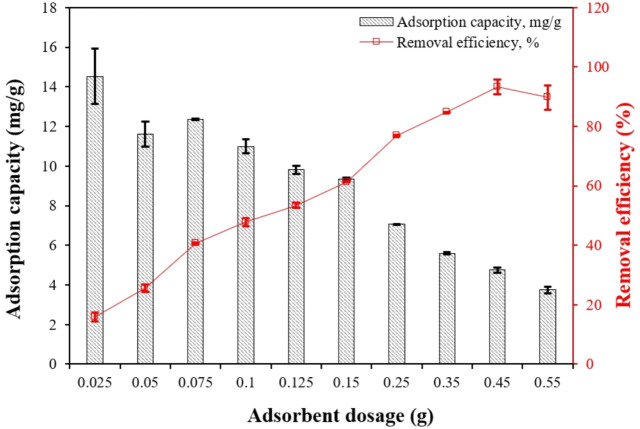
Effect of adsorbent dosage on PO_4_–P adsorption capacity (initial PO_4_–P concentration = 50 mg/L; solution volume = 50 mL; contact time = 30 min; initial pH = 5.198; solution temperature = 24.3 °C), mean ± SD.

The adsorption of PO_4_–P ions onto CS–CBC–Zr beads was studied at varying adsorbent dosages (0.025–0.55 g) without pH adjustment (Fig. 6). Adsorption was conducted for 30 min at 24 °C using an initial PO_4_–P solution of 50 mg/L. Phosphate ion removal steadily increased from 15.8% to 93.3% as the CS–CBC–Zr dosage increased from 0.025 to 0.45 g. This increase is likely due to the greater availability of more active sites for adsorption and a larger total surface area at higher CS–CBC–Zr dosages ^25^. A further increase in adsorbent dosage beyond 0.45 g did not remarkably affect removal efficiency. However, the adsorption capacity of CS–CBC–Zr decreased with increasing adsorbent dosages. As the CS–CBC–Zr dosage increased from 0.025 to 0.55 g, the amount of PO_4_–P adsorbed per gram of CS–CBC–Zr decreased from 14.5 to 3.74 mg/g. This effect is most likely due to some adsorption sites remaining unsaturated at higher dosages and the aggregation of adsorbent particles during the adsorption process ^62^. The optimal adsorbent dosage for PO_4_–P removal was determined to be 0.45 g, and this value was used in subsequent studies.

#### Effect of initial pH

**Fig. 7:**
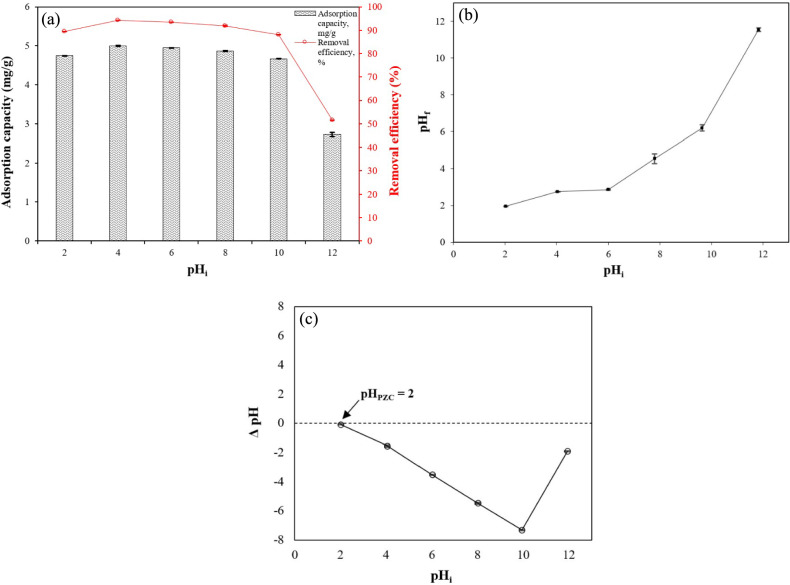
(**a**) Effect of pH on PO_4_–P adsorption (initial PO_4_–P concentration = 50 mg/L; solution volume = 50 mL; contact time = 30 min; dose = 0.45 g; solution temperature = 24.3 $^\circ$C); (**b**) solution pH after PO_4_–P adsorption; (**c**) determination of pH at the point of zero charge (pH_PZC_) for CS–CBC–Zr via the pH drift method, mean ± SD.

The effect of pH on the adsorption of PO_4_–P by CS–CBC–Zr was investigated at an initial PO_4_–P concentration of 50 mg/L and an adsorbent dosage of 0.45 g (= 0.9 g/L), and the results are shown in Fig. 7a. It was observed that as the initial solution pH increased from 4 to 10, the PO_4_–P adsorption capacity of CS–CBC–Zr slightly and steadily decreased from 4.99 to 4.67 mg/g. However, at a pH of 12, a sudden and significant decrease in PO_4_–P adsorption capacity was observed, declining to 2.73 mg/kg. This trend is likely due to the deprotonation of surface functional groups and the distribution of PO_4_–P species in the solution ^32^. At pH levels between 7 and 12, the predominant PO_4_–P species in the solution are in the form of HPO_4_^2−^, which are more difficult to adsorb than other phosphate forms due to their higher adsorption free energy ^64^. The increase in negative surface charge of the adsorbent with rising solution pH, combined with the greater negative charge of phosphate, results in strong electrostatic repulsion ^69^.

The removal efficiency of PO_4_–P by CS–CBC–Zr exceeded 90% in the pH range of 4–10. The highest removal efficiency (94.2%) was achieved at a pH of 4, leading to nearly complete PO_4_–P removal. A similar removal efficiency (95%) at pH 4.5 was reported in another study using hydrous zirconia-impregnated chitosan beads ^30^. When the pH increased to 10.0, the removal efficiency of PO_4_–P by CS–CBC–Zr remained sufficiently high (88.1%). This indicates that CS–CBC–Zr beads are effective over a wide pH range and demonstrates their structural and chemical stability under both strong acid and base conditions. However, at pH values exceeding 10, a significant decrease in adsorption capacity was observed. A similar trend was reported in another study using lanthanum-loaded cork biochar for PO_4_–P removal ^33^. This reduction in adsorption capacity was explained by the significant increase in OH^−^ concentration at a pH of > 10, which weakened ligand exchange interactions. At a high pH, Zr^4+^ binding to PO_4_^3−^ was inhibited under alkaline conditions. The presence of excess OH^–^ ions played a dominant role, competing with negatively charged PO_4_–P species for adsorption sites and further reducing the adsorption capacity of CS–CBC–Zr ^19^. The reduction in adsorption capacity at high pH levels could also be attributed to the weak electrostatic interactions between the anionic PO_4_–P species and CS–CBC–Zr. In alkaline conditions, the chitosan surface acquires a negative charge, which results in the electrostatic repulsion of anionic PO_4_–P species, preventing their adsorption ^70^.

Additionally, the final pH of the solution after PO_4_–P adsorption can provide insights into the adsorption mechanism. As shown in Fig. 7b, at an initial pH of 2 and after the adsorption of PO_4_–P ions, changes in the pH of the final solution were negligible and reached 1.96. When the initial pH of PO_4_–P solutions increased to pH 10, the final solution’s pH decreased. This occurred because the adsorption reaction resulted in a release of H^+^ ions, possibly due to the ionization of the Zr–OH groups ^71^.

The pH_PZC_ value of CS–CBC–Zr was measured to be approximately 2 (Fig. 7c). A pH_PZC_ of 2 indicates that the adsorbent surface is positively charged at a pH of < 2 and negatively charged at a pH of > 2. This suggests that when the solution’s pH is < pH_PZC_, the adsorbent surface remains positively charged, supporting the electrostatic attraction between the protonated adsorbent surface and PO_4_–P ions. However, when the solution’s pH exceeded 2, the adsorption capacity remained relatively stable up to a pH of 10. These results indicate that PO_4_–P adsorption primarily occurred via ion exchange rather than electrostatic attraction, consistent with previous findings ^72^. Chloride anions, which were bound to the CS–CBC–Zr surface, were replaced by phosphate anions, as confirmed by XPS analysis (“XPS analysis of adsorbents before and after adsorption”). The adsorbed phosphate formed strong inner-sphere complexes with zirconium centers (Zr–O–P) rather than being held by simple electrostatic attraction ^73^.

#### Effect of coexisting anions on adsorption

**Fig. 8:**
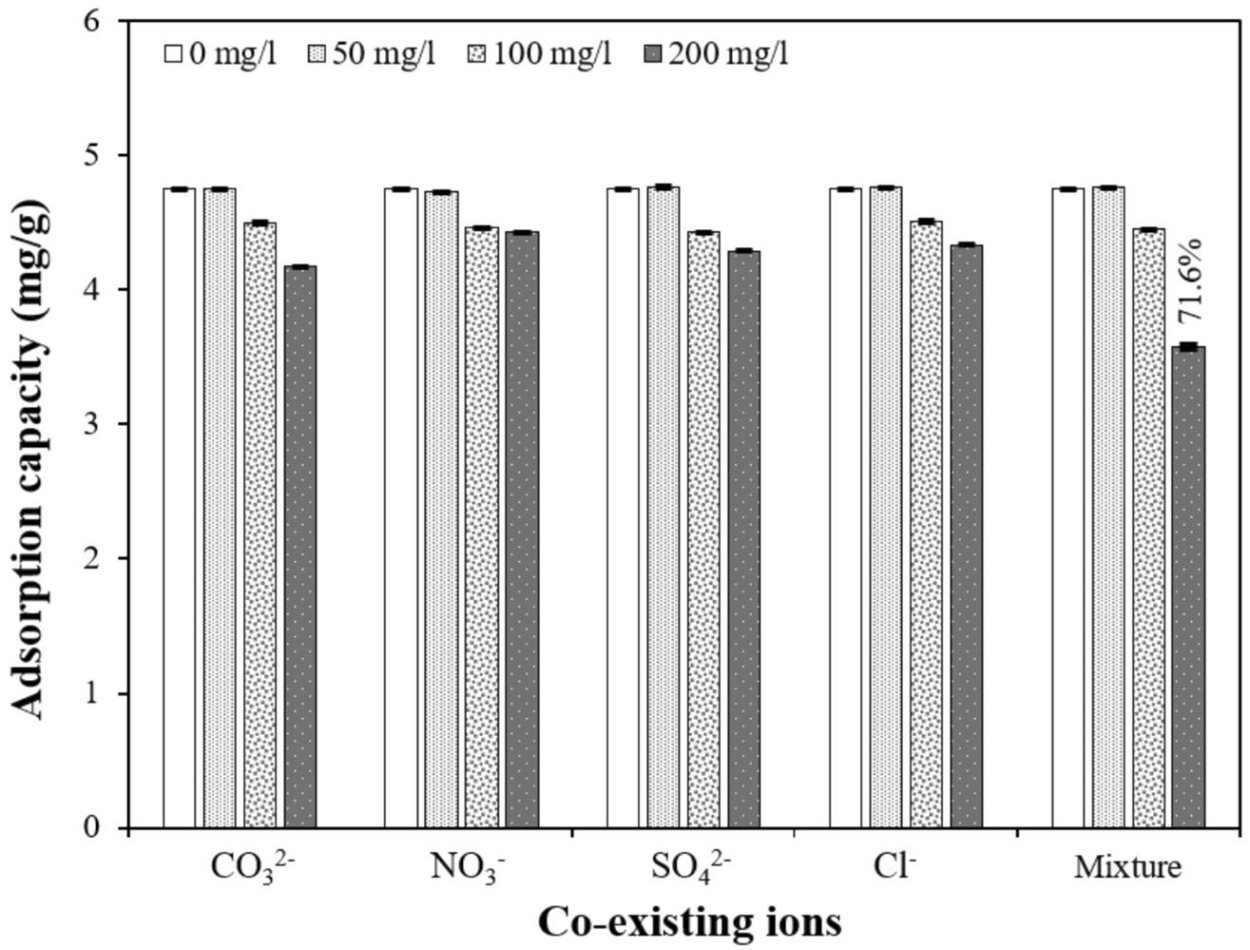
Influence of coexisting anions on PO_4_–P adsorption (initial PO_4_–P concentration = 50 mg/L; solution volume = 50 mL; contact time = 30 min; adsorbent dose = 0.45 g; initial pH = 5.144; solution temperature = 21.8 °C), mean ± SD.

The influence of coexisting anions (CO_3_^2–^, NO_3_^–^, SO_4_^2–^, Cl^–^, and their mixture) on PO_4_–P adsorption capacity was evaluated, and the results are presented in Fig. 8. When the concentrations of competing anions were 50 mg/L, CO_3_^2–^, NO_3_^–^, SO_4_^2–^, and Cl^–^ and their mixture had a negligible effect on PO_4_–P adsorption onto CS–CBC–Zr. This indicates that CS–CBC–Zr exhibits high selectivity and sensitivity to PO_4_–P. PO_4_–P removal efficiency remained around 95%, confirming the strong selectivity of CS–CBC–Zr for PO_4_–P anions. At a higher competing ion concentration (100 mg/L each anion), the presence of CO_3_^2–^, NO_3_^–^, SO_4_^2–^, Cl^–^, and their mixture reduced the PO_4_–P adsorption capacity of CS–CBC–Zr by 5–7%. Our findings align with previous research, demonstrating that other zirconium-based materials also exhibit strong selectivity for PO_4_–P^30,64^. However, when the concentration of all anions reached 200 mg/L, the PO_4_–P removal efficiency of CS–CBC–Zr decreased to 71.6% of its original value. This result indicates that the adsorption of PO_4_–P may be negatively affected in real wastewater systems that contain high concentrations of common coexisting anions competing for adsorption sites. To maintain effective PO_4_–P removal in such wastewaters, the increase in CS–CBC–Zr sorbent dosage may be necessary. Although the selectivity of adsorbent toward PO_4_–P is promising, future studies are needed to evaluate its performance in more complex conditions, such as real wastewater containing dissolved organic matter and heavy metals, which could also negatively affect the adsorption process.

### Adsorption isotherms and kinetics

#### Adsorption kinetics

**Fig. 9:**
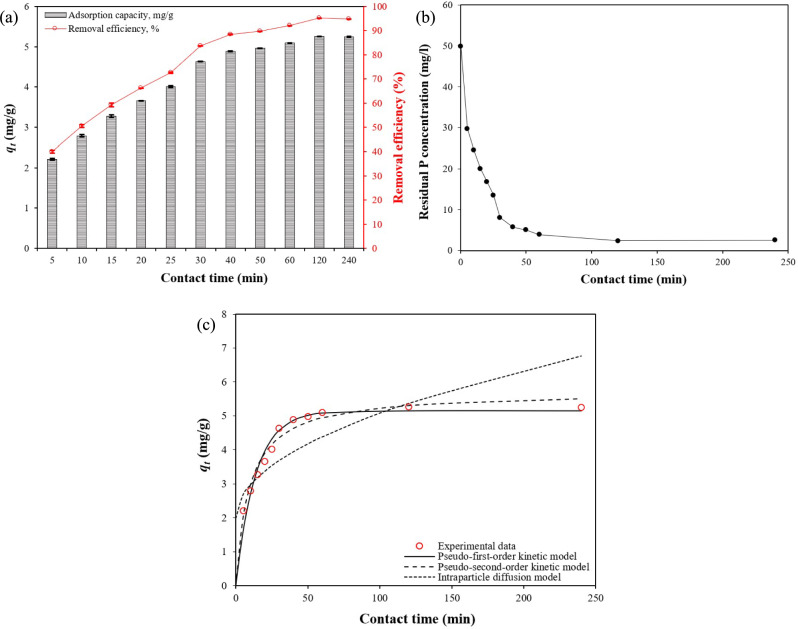
(**a**) Effect of contact time on PO_4_–P adsorption (initial PO_4_–P concentration = 50 mg/L; solution volume = 50 mL; adsorbent dose = 0.45 g; initial pH = 6.324; solution temperature = 21.8 °C), (**b**) residual PO_4_–P concentration, and (**c**) adsorption kinetic curves fitted using the pseudo-first-order, pseudo-second-order, and intraparticle diffusion models, mean ± SD.

The effect of contact time on PO_4_–P adsorption by CS–CBC–Zr was studied by varying the adsorption duration from 5 to 240 min. For the kinetic experiment, the initial PO_4_–P concentration was 50 mg/L, and the results are shown in Fig. 9a. Phosphates were rapidly adsorbed by CS–CBC–Zr beads during the initial stage of the adsorption process (0–30 min), reaching 88.07% of the equilibrium adsorption capacity within 30 min. This rapid adsorption rate was likely due to the uniform distribution of Zr ions on the chitosan–biochar surface, providing numerous readily accessible active sites for PO_4_–P adsorption. Within 120 min, the adsorption capacity reached equilibrium as the adsorption process stabilized, and the adsorption capacity remained relatively constant due to the saturation of all active adsorption sites with PO_4_–P. The time required to reach equilibrium was three times shorter compared to Zr-loaded magnetic biochar composites ^32^, and it was twice shorter compared to the zirconium-modified biochar ^64^. Similarly to adsorption capacity, the removal efficiency also increased over time. In the first 5 min, 40.01% of PO_4_–P was removed, and the removal efficiency gradually increased to 95.21% over a period of 120 min. Therefore, the optimum time required for PO_4_–P removal on CS–CBC–Zr was 120 min, and the PO_4_–P adsorption capacity of the adsorbent was equal to 5.26 mg/g.

To gain deeper insights into the adsorption process of PO_4_–P, the data shown in Fig. 9a were further analyzed using three adsorption kinetic models—pseudo-first-order, pseudo-second-order, and intraparticle diffusion models—as shown in Fig. 9c. The corresponding fitting parameters can be found in Table 1. The results indicate that both the pseudo-first-order and pseudo-second-order kinetic models accurately reproduced the experimental data. The experimental *q*_*e*_ value (5.26 mg/g) was close to the values calculated using the pseudo-first-order (5.15 mg/g) and pseudo-second-order (5.73 mg/g) kinetic models, further confirming the suitability of both models for describing PO_4_–P adsorption onto CS–CBC–Zr beads. However, between these two models, the pseudo-second-order kinetic model provided a better fit to the adsorption kinetics, as evidenced by its higher coefficient of determination (R^2^ = 0.959 > 0.943) and lower values for all error functions. This suggests that the pseudo-second-order model more accurately represented the actual adsorption process (Table 5). These findings indicate that the overall adsorption process was dominated by chemical adsorption ^30,74^. These results were consistent with previous studies on PO_4_–P adsorption onto other zirconium-modified adsorbents ^23,47^. The intraparticle diffusion model exhibited the worst fit, as indicated by the lowest coefficient of determination (R^2^ = 0.487). Thus, based on the fitting parameters, it can be concluded that the primary adsorption mechanism was chemical adsorption, while the secondary mechanism was physical adsorption.

**Table 5:** Error function values and coefficient of determination (R^2^) for the pseudo-first-order, pseudo-second-order, and intraparticle diffusion models.

Model	Error function					
	SSE	HYBRID	MPSD	RMSE	${\text{x}}^{2}$	R^2^
Pseudo-first-order	0.655	2.513	1.257	0.244	0.328	0.943
Pseudo-second-order	0.458	1.214	0.607	0.205	0.123	0.959
Intraparticle diffusion	5.814	12.43	6.213	0.729	1.270	0.487

#### Adsorption isotherms

**Fig. 10:**
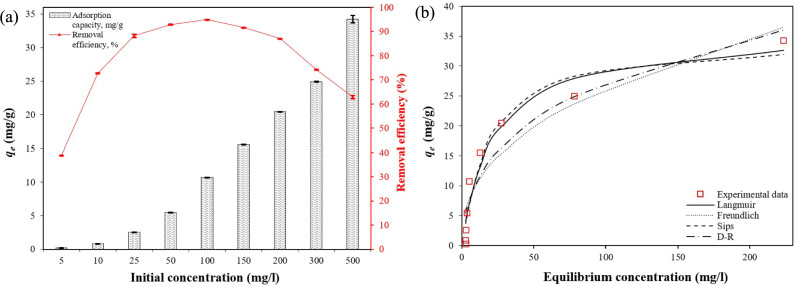
(**a**) Effect of the initial PO_4_–P ion concentration on PO_4_–P adsorption capacity and removal efficiency (adsorbent dose = 0.45 g; contact time = 120 min; solution volume = 50 mL; initial pH = 6.350; solution temperature = 21.8 °C); (**b**) Langmuir, Freundlich, Sips, and Dubinin–Radushkevich isotherm models for PO_4_–P adsorption, mean ± SD.

For the adsorption equilibrium studies, the initial PO_4_–P concentrations ranged from 5 to 500 mg/L, with an adsorbent dose of 0.45 g and an optimized contact time of 120 min (Fig. 10a). It was observed that the adsorption capacity of PO_4_–P ions gradually and significantly increased with an increase in the initial concentration. When the initial concentration reached 500 mg/g, the attained *q*_*e*_ value was the maximum value, and it reached 34.2 mg/g.

Additionally, the Langmuir, Freundlich, Sips, and D–R isotherm models were used to describe the PO_4_–P adsorption process on CS–CBC–Zr. The adsorption isotherms are displayed in Fig. 10b, with fitting parameters listed in Table 1 and error function values shown in Table 6. Based on Table 6, the Sips and Langmuir isotherm models effectively fitted the isotherm data, achieving R^2^ values higher than 0.94. These results suggest that the Sips and Langmuir isotherm models better describe the relationship between *C*_*e*_ and *q*_*e*_ than the Freundlich and D–R models. However, the Sips model demonstrated superior performance, with the highest R^2^ (0.944) and the lowest error values compared to the other models. Similar results have been reported when other Zr-loaded adsorbents were used for PO_4_–P adsorption ^63^. The calculated *R*_*L*_ value was 0.043 for the highest studied initial adsorbate concentration (500 mg/L), which was lower than 1 (Table 1). According to adsorption theory, *R*_*L*_ values lower than 1 indicate favorable adsorption conditions ^17^. It was also found that the *R*_*L*_ value gradually decreased as the initial PO_4_–P concentration increased (e.g., *R*_*L*_ = 0.816 at 5 mg/L, *R*_*L*_ = 0.308 at 50 mg/L, *R*_*L*_ = 0.043 at 500 mg/L). This trend indicates that at lower PO_4_–P concentrations, *R*_*L*_ was closer to 1, suggesting less favorable conditions for adsorption. Similarly to *R*_*L*_, the 1/*n* value obtained from the Freundlich model (0.415) also confirmed favorable adsorption conditions, as adsorption theory states that a 1/*n* value between 0 and 1 indicates favorable adsorption ^17^. The observed index *n* (1.124) from the Sips model was close to 1, further exhibiting highly homogeneous and favorable adsorption ^75^. These findings suggest that the PO_4_–P adsorption onto CS–CBC–Zr occurred through a monolayer, homogenous adsorption process, with no further adsorption once saturation was reached ^76^. According to the D–R isotherm, the calculated mean free energy of adsorption (*E* = 11.24 kJ/mol) indicates that the adsorption of PO_4_–P onto the surface of CS–CBC–Zr beads is chemical in nature and occurs via ion exchange (Table 1). Based on the free energy of adsorption values, findings from other studies also suggest that phosphate adsorption onto Zr-modified materials is primarily governed by a chemisorption mechanism involving ion exchange reactions ^23,62^.

**Table 6:** Error function values and coefficient of determination (R^2^) for the Langmuir, Freundlich, Sips, and Dubinin–Radushkevich isotherm models.

Model	Error functions					
	SSE	HYBRID	MPSD	RMSE	${\text{x}}^{2}$	R^2^
Langmuir	63.49	1198	598.8	2.687	10.49	0.943
Freundlich	138.8	2559	1279	3.928	17.45	0.878
Sips	63.49	992.3	496.2	2.656	9.959	0.944
D-R	109.1	2125	1063	3.482	14.99	0.904

By analyzing R^2^ and error function values, the best fit was observed in the Sips model (R^2^: 0.944; SSE: 63.49; RMSE: 2.656) (Table 6). The suitability of the Sips isotherm demonstrated that the adsorption process on the heterogeneous CS–CBC–Zr surface exhibited homogeneous behavior. The Sips exponent value (1.124) indicated homogeneous adsorption with a slight tendency towards cooperative effects. This suggests the possibility of cooperative adsorption, where the adsorption of one phosphate ion enhances the adsorption of subsequent ions. The maximum adsorption capacity (*q*_*max*_) predicted by the Sips model (33.89 mg/g) was lower than that predicted by the Langmuir isotherm (35.94 mg/g), yet it showed closer agreement with the experimental value (34.25 mg/g). The Sips model is considered to provide a more realistic estimation of adsorption capacity due to its ability to account for surface heterogeneity ^77^.

#### Performance comparison with other adsorbents

The maximum PO_4_–P adsorption capacity is a key factor in determining the potential efficiency and performance of sorbents. The *q*_*max*_ values for PO_4_–P achieved by other zirconium-based adsorbents are presented in Table 7. The *q*_*max*_ estimated by the Sips model in this study was 33.89 mg/g, which was comparable to or significantly higher than other Zr-modified adsorbents previously reported in the literature. Notably, the *q*_*max*_ value obtained in this study is nearly four times higher than zirconium-loaded lignocellulosic butanol residue and twice higher than that of zirconium hydroxide ^78,79^. However, it is approximately two times lower than that obtained for Zr-modified chitosan beads in a previous study ^63^. This could be explained by the larger particle size of our adsorbent (1.74 mm) compared to the zirconium-modified chitosan beads (20–60 µm) ^63^. In addition, Zr content in our composite was substantially lower, which resulted in a lower amount of the active sites available for phosphate adsorption. Although CS–CBC–Zr beads exhibited a lower PO_4_–P adsorption capacity compared with zirconium-modified chitosan beads, the inclusion of biochar derived from used cork stoppers enhances the sustainability of composite material by utilizing low-cost, renewable byproducts of the cork industry.

**Table 7:** Comparison of maximum adsorption capacity (*q*_*max*_, mg P/g) with other reported zirconium-based adsorbents.

Adsorbent	Initial concentation (mg/L)	Dosage (g/L)	pH	Contact time (min)	*q*_*max*_ (mg/g)	References
Zr-loaded lignocellulosic butanol residue	–	0.625	6	2880	8.75	^78^
Zirconium hydroxide	10	0.1–3	7.1	4320	18.5	^79^
Zr-modified calcium montmorillonite	1–50	0.025	4–10	1440	22.37	^80^
Zr-modified activated sludge	5–50	0.5	–	360	27.55	^81^
Zr-impregnated chitosan beads	0–150	0.75	6.7	5760	42.02	^30^
Amino-functionalized magnetic zirconium alginate beads	100–150	2.5	2	1800	42.23	^82^
Zr-loaded okara	10–500	0.5	–	1440	14.4	^25^
Zr-loaded magnetic chitosan/poly(vinyl alcohol) hydrogel	1–30	0.25	6.5	1440	50.76	^72^
Zr-modified peanut shell biochar	0.5–100	0.1	–	240	19.21	^64^
Zirconium oxide embedded in quaternary-ammonium Chinese reed	20–200	1	5.4	1440	59.2	^73^
Chitosan beads modified by zirconium	5–50	0.2	4	0–180	60.6	^63^
Zr-loaded cross-linked chitosan particles	10–300	0.1	3–7	5–100	23.37	^62^
Chitosan modified Zr-loaded CBC beads	5–500	9	6.4	120	33.89	This work

### Desorption study and reusability

**Fig. 11:**
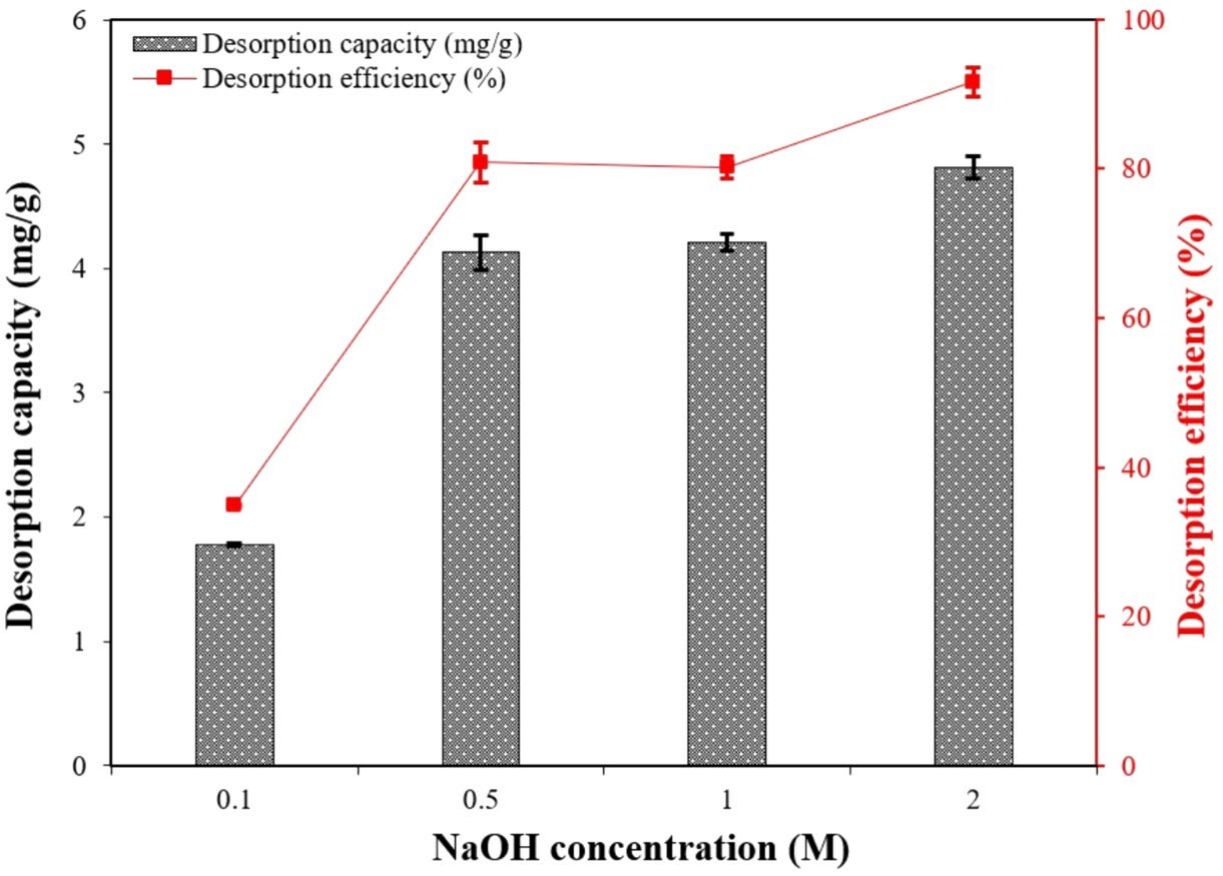
Desorption capacity (mg/g) and desorption efficiency (%) of PO_4_–P adsorbed on CS–CBC–Zr beads using different concentrations of NaOH solutions (adsorbent dose = 0.45 g; contact time = 60 min; solution volume = 50 mL), mean ± SD.

A desorption study is useful for understanding adsorption mechanisms and optimizing processes to enhance cost-effectiveness. For PO_4_–P desorption and CS–CBC–Zr bead regeneration, several ion exchange solutions (0.1 M NaCl and 0.1 M NaOH) were evaluated, and the most effective desorbent was selected for further regeneration experiments. The first experiment showed that composite beads immersed in a 0.1 M NaOH solution exhibited a desorption capacity (*q*_*d*_) of 1.74 mg/g, whereas beads immersed in a 0.1 M NaCl solution exhibited 0 mg/g. This result confirmed that the NaCl solution had no significant desorption effect, which could be attributed to electrostatic adsorption and ligand exchange mechanisms ^32^. The positive desorption effect of the NaOH solution could be related to the fact that alkaline solutions (such as NaOH) release a high concentration of hydroxide ions, which interact with PO_4_–P ions. These hydroxide ions disrupt the chemical bonds between the adsorbent and PO_4_–P, causing PO_4_–P desorption and release into the solution ^83^. However, 0.1 M NaOH exhibited a desorption efficiency of only 35%, and this is likely due to the short desorption time or low desorbent concentration. To further investigate the impact of NaOH concentration on PO_4_–P desorption, an additional experiment was conducted using 0.1 M, 0.5 M, 1 M, and 2 M NaOH solutions. The desorption efficiency exhibited a significant (*p* < 0.05) improvement as NaOH concentrations increased from 0.1 M to 2 M (Fig. 11). However, there was no significant change in desorption efficiency between 0.5 M and 1 M NaOH solutions (*p* = 0.8). Since a significant increase (*p* = 0.02) in desorption efficiency was observed between 1 and 2 M NaOH, desorption using the 2 M NaOH solution was repeated to test material reusability. After the first adsorption–desorption cycle using 2 M NaOH, the achieved regeneration efficiency for the composite beads reached 40%. The relatively low regeneration efficiency suggests that changes in the chemical structure of the adsorbent surface may have occurred, possibly due to the dissolution of loaded Zr from the composite beads by the NaOH desorption solution, thereby reducing their ability to re-adsorb phosphate. Therefore, the reusability of these materials would be limited in water treatment applications that require several adsorption–desorption cycles of the sorbent.

### Proposed mechanisms for phosphate adsorption

**Fig. 12:**
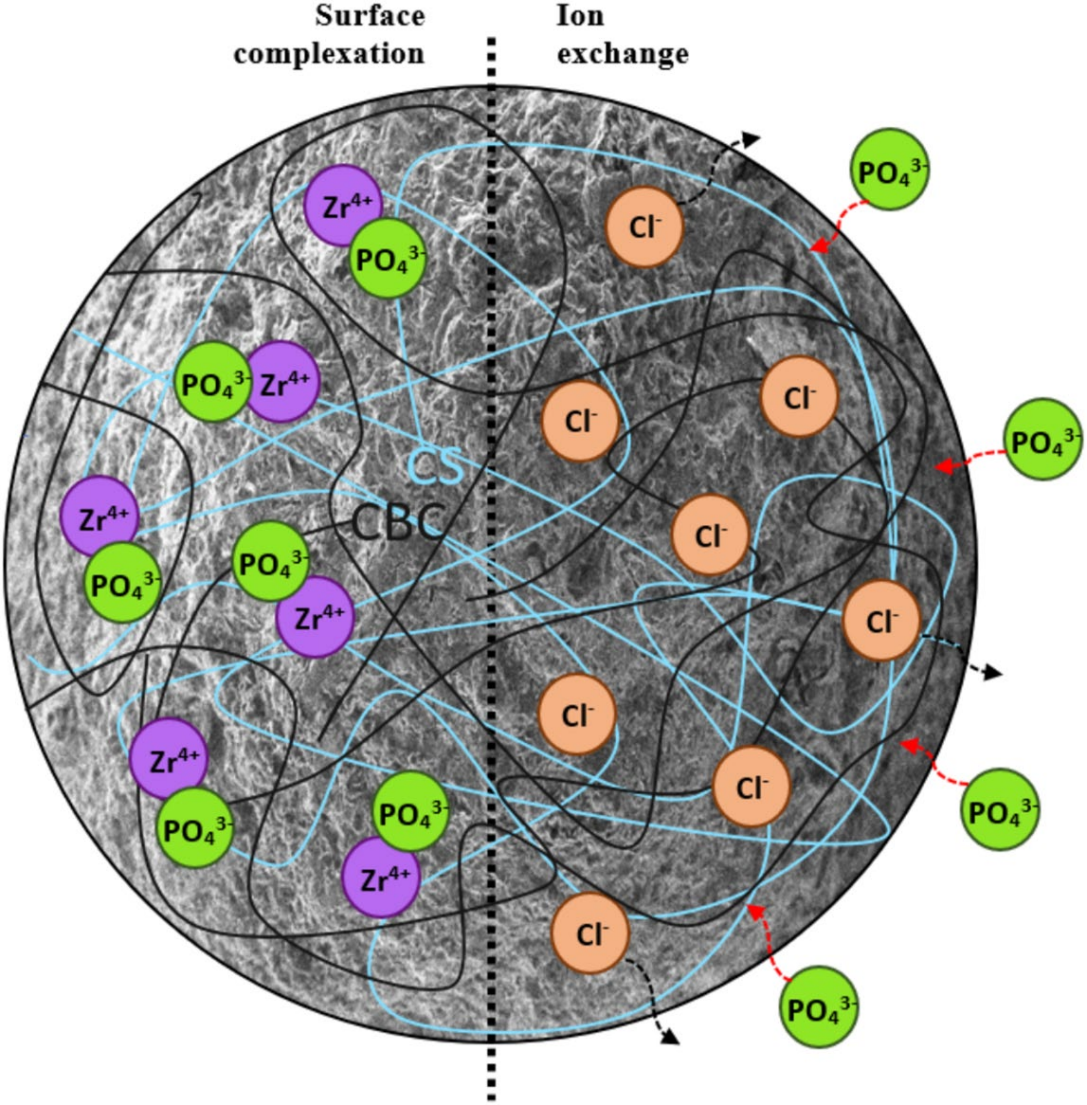
Proposed mechanisms of PO_4_–P adsorption by CS–CBC–Zr.

In this study, the adsorption of PO_4_–P onto CS–CBC–Zr beads was analyzed through batch adsorption experiments, wherein the effect of ZrOCl_2_·8H_2_O concentration was analyzed, alongside FTIR analysis and kinetic modelling data. According to the literature, Zr(IV) ions can interact with phosphate ions through ion exchange, electrostatic attraction, and the formation of surface-bound complexes ^47,64^. In this study, the possible mechanisms of PO_4_–P adsorption included inner-sphere complexation and ion exchange. The FTIR analysis results were particularly important for explaining complexation mechanisms. Phosphate binding to CS–CBC–Zr beads was observed, as evidenced by a characteristic peak at 1072 cm^−1^, which slightly decreased after adsorption, suggesting the formation of Zr–O–P complexes. Additionally, the peak at 1437 cm^−1^ diminished, indicating an exchange between OH and P in the solution. The results of the point-of-zero-charge (pH_PZC_) experiment demonstrated that electrostatic attraction was not the main mechanism in phosphate adsorption and that the primary mechanism responsible for PO_4_–P adsorption was instead Cl^−^ exchange. This was also confirmed by XPS analysis ("XPS analysis of adsorbents before and after adsorption"). Based on the experimental results discussed above, the proposed mechanisms for PO_4_–P adsorption onto CS–CBC–Zr are illustrated in Fig. 12. Some previous studies on the mechanism of PO_4_–P adsorption onto Zr-based composites indicated that the primary process involved the substitution of the hydroxyl group attached to zirconium (Zr–OH) with P ^71^, resulting in the formation of Zr–O–P inner-sphere complexes; this is the dominant adsorption mechanism when the pH exceeds the pH_PZC_.

According to Fig. 5, it is evident that zirconium(IV) played a primary role in PO_4_–P adsorption onto the CS–CBC–Zr beads, as the adsorption capacity for PO_4_–P ions increased approximately sixfold compared to CS–CBC (with crosslinker) beads (*p* = 0.003). Simultaneously, the crosslinker itself had no significant impact on PO_4_–P adsorption capacity (*p* = 0.7). Other studies have also shown that zirconium loading on the adsorbent is the primary factor influencing PO_4_–P adsorption. For example, Wan et al. ^72^ found that the maximum PO_4_–P adsorption capacity on hydrogels (10.11 mg/g) was only 20% of that obtained on Zr-loaded hydrogels. Correspondingly, Zong et al. ^78^ found that pure lignocellulosic butanol residue (LBR) without Zr loading exhibited negligible PO_4_–P adsorption (1.92 mg/g), which was 4.56 times lower than that of Zr-loaded LBR (8.75 mg/g).

## Conclusions

In this study, a novel adsorbent, CS–CBC–Zr composite beads, was successfully developed for the removal of various concentrations of PO_4_–P using cork stopper biochar as a support material. The synthesis involved chitosan and biochar gel preparation, followed by chemical crosslinking and subsequent Zr loading. Zirconium loading at 3% was found to be the most effective in enhancing the active sites of the CS–CBC–Zr composite beads, thereby improving adsorption performance. The adsorption of phosphate ions onto CS–CBC–Zr beads was best described by the Sips isotherm (n = 1.124, R^2^ = 0.94), indicating a certain degree of surface heterogeneity and adsorption behavior approaching monolayer coverage. A mean free energy value of 11.24 kJ/mol suggests that phosphate adsorption onto CS–CBC–Zr is primarily governed by chemisorption, characterized by ion exchange reactions at active sites. The maximum adsorption capacity was calculated to be 33.89 mg/g based on the Sips model, which was very close to the experimental value. The equilibrium time for PO₄–P adsorption onto CS–CBC–Zr beads was 120 min. Stable performance across a wide pH range (4–10) and notable phosphate selectivity were also observed. Structural changes in the composite beads at both physical and chemical levels were confirmed by SEM, XPS, and FTIR analyses. The findings of the study show that CS–CBC–Zr exhibited strong capability in removing various PO_4_–P concentrations, presenting a promising approach not only for mitigating water eutrophication but also for treating heavily polluted wastewater. Future studies should focus on the application of CS–CBC–Zr composite adsorbents in wastewater treatment for the removal of diverse pollutants. Investigating their potential for adsorbing other inorganic and organic contaminants, such as heavy metals and dyes, could offer valuable insights and significantly advance this research area by broadening the practical applications of these composites.

## Data Availability

The data supporting the findings of this study can be obtained from the corresponding author on a reasonable request.
